# Transitional Polytherapy: Tricks of the Trade for Monotherapy to Monotherapy AED Conversions

**DOI:** 10.2174/157015909788848884

**Published:** 2009-06

**Authors:** William R Garnett, Erik K St. Louis, Thomas R Henry, Thomas Bramley

**Affiliations:** 1 Virginia Commonwealth University, Richmond, Virginia, USA; 2 Mayo Clinic, Rochester, Minnesota, USA; 3 University of Minnesota, Minneapolis, Minnesota, USA; 4 Xcenda, Salt Lake City, Utah, USA

**Keywords:** Epilepsy, antiepileptic drugs, titration, conversion, monotherapy, polytherapy, pharmacokinetics.

## Abstract

The goal of epilepsy therapy is to help patients achieve seizure freedom without adverse effects. While monotherapy is preferable in epilepsy treatment, many patients fail a first drug due to lack of efficacy or failure to tolerate an initial medication, necessitating an alteration in therapy. Sudden changes between monotherapies are rarely feasible and sometimes deleterious given potential hazards of acute seizure exacerbation or intolerable adverse effects. The preferred method for converting between monotherapies is transitional polytherapy, a process involving initiation of a new antiepileptic drug (AED) and adjusting it toward a target dose while maintaining or reducing the dose of the baseline medication. A fixed-dose titration strategy of maintaining the baseline drug dose while titrating the new medication is preferable when breakthrough seizures are occurring and no adverse effects are present. However, a flexible titration strategy involving reduction of the baseline drug dose to ensure adequate tolerability of the new adjunctive medication is preferred when patients are already experiencing adverse effects. This article reviews pharmacokinetic considerations pertinent for ensuring successful transitional polytherapy with the standard and newer antiepileptic drugs. Practical consensus recommendations “from an expect panel (SPECTRA, Study by a Panel of Experts Considerations for Therapy Replacement and Antiepileptics) for a successful monotherapy” AED conversions are then summarized. Transitional polytherapy is most successful when clinicians appropriately manage the titration strategy and consider pharmacokinetic factors germane to the baseline and new adjunctive medication.

## INTRODUCTION

Epilepsy is characterized by two or more unprovoked seizures that may be either partial (focal or localized) or generalized. A single seizure does not define epilepsy because the probability of having a second seizure ranges between 20% and 70%. Therefore, depending on the underlying risk factors, therapy with antiepileptic drugs (AEDs) is usually not initiated until a patient has a second seizure. However, when it is established that a patient has epilepsy (i.e., two or more unprovoked seizures), rapid initiation of therapy to control seizures is appropriate.

The goal of seizure therapy is producing seizure freedom without adverse effects of treatment. The choice of the initial AED involves selecting a drug that is appropriate for the patient’s seizure type, the safety profile for that individual and the patient’s ability to tolerate the drug, the potential for interactions with other drugs, the pharmacokinetics and pharmacodynamics that determine the dosing schedule, a formulation that the patient can ingest, and the expense.

While monotherapy is preferable in epilepsy treatment, many patients fail a first drug due to lack of efficacy or failure to tolerate an initial medication, necessitating an alteration in therapy. Sudden changes between monotherapies are rarely feasible and sometimes deleterious since they expose the patient to potential hazards of acute seizure exacerbation or intolerable adverse effects. The preferred method for converting between monotherapies is transitional polytherapy, a process involving initiation of a new antiepileptic drug (AED) and adjusting it toward a target dose while maintaining the baseline medication. A flexible titration strategy involving reduction of a baseline drug dose to ensure adequate tolerability of the new adjunctive medication is preferred. This article reviews pharmacokinetic considerations pertinent for ensuring successful transitional polytherapy with the standard and newer antiepileptic drugs. Practical consensus recommendations from an expert panel for successful monotherapy to monotherapy AED conversions are then summarized. Transitional polytherapy is most successful when clinicians appropriately manage the titration strategy and consider pharmacokinetic factors germane to the baseline and new adjunctive medication.

## MONOTHERAPY

A guiding principal of AED therapy is the initiation of therapy with a single AED (ie, monotherapy). While AED therapy formerly was often initiated with combination therapy (eg, phenytoin and phenobarbital), the principle of monotherapy was established in the 1980s, based on studies that demonstrated comparable efficacy and superior tolerability of monotherapy compared to polytherapy. Initial therapy of newly diagnosed epilepsy with combination AEDs is inappropriate because most patients respond to a single AED [27,28]. The use of a second AED increases a patient’s drug load, adding to the number and severity of side effects. A second drug also increases the potential for drug-drug interactions and makes drug regimens more complex. More complex drug regimens result in a lower rate of compliance (adherence) [9]. Other potential limitations of initial AED polytherapy are that if two AEDs are used initially, it is difficult to titrate each drug individually, and the use of combination therapy is more expensive. Therefore, while combination therapy may be appropriate in many refractory patients in chronic epilepsy treatment, it is not appropriate as initial therapy. Many clinicians feel that polytherapy is not appropriate until a patient has failed two or three trials of monotherapy with appropriately selected AEDs for that patient’s epilepsy syndrome.

A factor that limits the use of monotherapy with some newer AEDs is the stringent FDA approval process for monotherapy treatment in epilepsy, which requires demonstration of superiority to another treatment, usually arising from a large randomized controlled trial utilizing an active control design. AEDs may be approved for use in some countries following equivalency trials. In these trials, the new AED will be compared to an established AED such as carbamazepine. If a new drug is found to be at least as effective as the standard, the drug is approved following proof of equivalency or non-inferiority. The FDA viewpoint is that while drugs may be equivalent, they may be equally ineffective. In some studies, a low and presumably ineffective dose of a standard AED, such as valproic acid, was used in place of the placebo. While a placebo-controlled study is considered the “gold standard” for proof of principle for efficacy in clinical trials, most experts would consider treatment of newly diagnosed epilepsy with a placebo to be unethical. Therefore, all of the newer or “second-generation” AEDs beginning with the approval of felbamate in 1993 have been initially studied as add-on therapy for patients with uncontrolled partial seizures with or without secondary generalization. Entry criteria for such studies typically require continuing seizures despite therapy with one to three AEDs in adequate doses. Therefore, initial efficacy is determined as combination therapy.

Drugs that are efficacious as combination therapy may not be efficacious as monotherapy. A drug which is proven to be efficacious for seizure reduction when given as adjunctive therapy may only be effective due to a pharmacodynamic combined effect with the original baseline drug; that is, the second AED may require the additional seizure suppressive activity of the first drug to be effective. There are several potential clinical trial designs for evaluating the efficacy of an AED for use as monotherapy depending on its pharmacokinetics and pharmacodynamics. One of the most frequently used designs is a so-called conversion to monotherapy design, in which the patient is initially treated with combination therapy, and then the initial AED is discontinued to assess the efficacy of the new drug alone. A few of the second-generation AEDs have conducted trials to demonstrate efficacy as monotherapy. For example, lamotrigine, topiramate, oxcarbazepine, and felbamate have been demonstrated to be effective as monotherapy and have received FDA approval for use as monotherapy, while gabapentin also has evidence for monotherapy use [18]. A recent study has also documented the efficacy of levetiracetam monotherapy efficacy in large comparator trial designs [7].

The initial choice of an AED has little effect on the response rate as long as the AED is appropriate for the seizure type [28]. In an assessment of a large number of drug naïve patients with newly diagnosed epilepsy, almost 50% of patients will respond to an AED regardless of which drug is utilized [28]. Thus, factors such as tolerability, safety, dosing regimen, and expense are more important in selecting an AED. However, if 50% of patients respond to initial therapy, this implies that another 50% do not respond to an initial AED. Many clinicians feel that patients should fail two or three trials of monotherapy before chronic polytherapy is entertained.

A sudden overnight switch from one AED to another is ill advised, given the efficacy of the newly planned AED has not yet been established, placing the patient at risk for catastrophic seizure worsening, raising the risk for acute repetitive seizures or even status epilepticus. Conversely, patients may not tolerate a rapid titration to target dose of the newly planned AED even if it is effective from its inception. Most clinicians agree that a gradual conversion process to a new planned AED monotherapy is the safest and most tolerable approach, a process that may be called transitional polytherapy. Transitional polytherapy involves a gradual titration of the newly planned AED toward a target dose, and, after this is achieved, a gradual withdrawal of the baseline drug. During this process, if intolerable adverse effects emerge, accelerating the withdrawal of the baseline initial drug may be considered.

Patients who fail to respond to their initial AED will need transitional polytherapy during conversion to a new monotherapy with another AED. Another reason requiring transition to a new medication is that a patient may be unable to tolerate their initial medication, irrespective of clinical effectiveness of that therapy. Such patients also need transitional polytherapy during a switch to another new AED monotherapy. Therefore, patients who fail to achieve seizure control, and/or are unable tolerate an initial AED, have need for transitional polytherapy.

## TRANSITIONAL POLYTHERAPY DURING CONVERSION FROM MONOTHERAPY TO MONOTHERAPY

There are many reasons why a given patient may not tolerate a specific AED. Some patients have idiosyncratic reactions necessitating removal of a drug. For example, phenytoin, carbamazepine, and lamotrigine are associated with allergic rash. In some cases, this rash may progress to a life-threatening condition such as the Stevens-Johnson syndrome or toxic epidermal necrolysis (TEN). More commonly, adverse effects may not be acute or life threatening but are troublesome to the patient. For example, some patients note diplopia, blurred vision, drowsiness, and lethargy with carbamazepine. While these CNS adverse effects may be reduced with a controlled release formulation of carbamazepine or a lower dose, the effects may still limit a patient’s quality of life (QoL). There is a negative linear relationship between adverse effects and patient QoL: as adverse events increase, patient QoL decreases [20]. In women of childbearing potential, teratogenic effects of specific AEDs are of concern. Several pregnancy registries have raised concerns regarding the use of valproic acid in women of child bearing potential [4,48,50]. Other patients have difficulty adhering to a specific drug regimen, especially regimens involving multiple daily doses; if the AED has to be given three or four times per day, adherence greatly diminishes [9]. Also, some patients, especially children and the elderly, may find that they cannot ingest certain AED formulations. These patients may need to be switched to a drug that is available as a sprinkle, liquid, or smaller pill size. Finally, changes in financial status or insurance coverage may make acquisition of several AEDs difficult.

The conversion of a patient on monotherapy with an initial baseline AED to a new planned AED may be rapid or slow. A rapid conversion would involve abruptly stopping the initial baseline drug and starting the newly planned drug. This is usually only done in a situation where the person has experienced a life-threatening reaction to an AED. The sudden withdrawal of an AED may precipitate a withdrawal seizure. Generally, a patient will be more slowly converted from an initial baseline AED to a new planned AED so that there is a transitional period of polytherapy. One method for a slow conversion is to begin to slowly reduce the dose of an initial baseline AED and to initiate a titration process with a new planned AED. Another method, generally preferred especially in patients who are tolerating an initial baseline drug but who have not achieved seizure control, maintains the dose of the baseline drug while the dose of the planned drug is titrated to the desired amount. Then the baseline drug is tapered off.

**Fig. (1):**
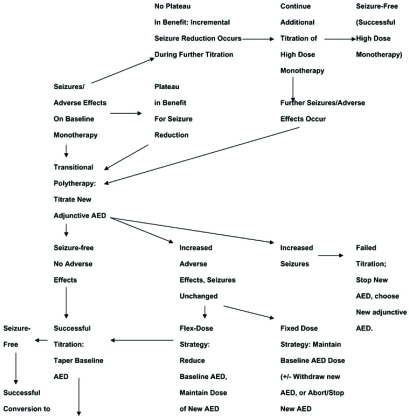
**Suggested Algorithmic Approach for Initiating and Carrying Out Transitional Polytherapy.** There are two potential pathways for using the algorithm which consider the alternative common clinical scenarios of either breakthrough seizures (beginning in upper left corner) or adverse effects (lower right corner) during initial monotherapy. If a patient experiences breakthrough seizures without adverse effects on monotherapy, the algorithm suggests further titration of high dose monotherapy until a “therapeutic plateau” (i.e., a maximal level of seizure reduction response) is reached, then progressing to a “Fixed-dose” transitional polytherapy strategy. Alternatively, if the patient experiences adverse effects, a “Flex-dose” strategy is most appropriate, which involves simultaneous reduction and tapering of the primary baseline antiepileptic drug (AED) while titrating the new adjunctive AED. The anticipated successful outcomes of these strategies would be seizure freedom on high dose monotherapy, seizure freedom following conversion to a new monotherapy, or a trial of chronic maintenance polytherapy with both the baseline and adjunctive AED.

The relative merits of further titration of the current drug toward the goal of high dose monotherapy, or alternatively initiating transitional polytherapy, is a central aspect of epilepsy care. Unfortunately, there is no evidence to guide this decision point, making the process more art than science (please see suggested algorithmic approach toward pursuit of high dose monotherapy *vs*. initiation of polytherapy shown in (Fig. **1**)). In newly diagnosed epilepsy, approximately 50% of patients respond to the first AED utilized, and the majority of patients respond to moderate target doses [26,27]. Exceptional patients clearly benefit from further vigorous titration of initial AED monotherapy. For patients that demonstrate incremental improvements in seizure frequency as AED dosing is increased without reaching a plateau in therapeutic effect, additional titration of high dose AED monotherapy is entirely reasonable and preferable as the logical goal of epilepsy care is to produce seizure freedom. Occasional patients continue to show improvement, and rarely become seizure free, when their AED therapy is dosed well beyond the average effective dose for that drug. The only limitation in such scenarios is avoiding intolerable dose-related AED adverse effects such as sedation, cognitive slowing, and ataxia.

However, for the majority of patients, further aggressive titration beyond an average effective AED dose is a largely futile enterprise yielding only additional adverse effects and little improvement in seizure burden. As such, it is reasonable to define treatment failure and the practical endpoint of initial monotherapy as continued breakthrough seizures occurring despite employing an average effective daily AED dosage. Such a patient is evolving toward refractory epilepsy even at this point. It is then reasonable and appropriate to begin a new adjunctive AED with the plan of converting to a second monotherapy, necessitating at least a brief period of transitional polytherapy.

There are then several possible outcomes and logical courses of further treatment (Fig. **1**): (1) the patient becomes seizure free when the new AED is added and remains seizure free with ensuing taper of the primary baseline AED; this patient is a successful monotherapy conversion and is treated with the newly added drug in monotherapy; (2) the patient becomes seizure free on the combination of AEDs during transitional polytherapy, has further disabling seizures during attempted taper of the initial baseline AED, and becomes seizure free again when the baseline primary drug is increased in dose or again reinstituted; this patient is thus best treated with a combination of both drugs in chronic maintenance polytherapy; 3) the patient continues having seizures despite the combination therapy, and seizure frequency actually worsens and/or intolerable adverse effects develop during titration of the new adjunctive AED; in this instance, resumption of the initial AED and another attempted adjunctive AED titration can be offered.

Alternatively, the main problem with intitial monotherapy may be adverse effects. Typical dose-related adverse effects include sedation, ataxia, and cognitive slowing. In this situation, options include dose reduction of the antiepileptic drug provided the patient remains seizure free, or employing a new adjunctive AED in transitional polytherapy with the goal of converting the patient over to that drug as a new second monotherapy. In this situation, adopting a “Flex-dose” strategy of drug titration (i.e., titrating the new adjunctive AED while tapering the primary baseline AED) is usually more successful in a patient who is already experiencing unpleasant dose-related adverse effects, which are likely to become exacerbated further by treatment with two drugs; for this reason, it is most reasonable to being a taper of the primary AED during the period of drug overlap. Potential outcomes include successful conversion to a new monotherapy with the new drug, or chronic maintenance polytherapy should the patient continue to have breakthrough seizures with attempted withdrawal of the primary baseline AED. The reader is again referred to (Fig. **1**) for a summary of this approach, and for further detailed discussion of polytherapy to the next article in this series entitled “Truly “Rational” Polytherapy: Maximizing Efficacy and Minimizing Drug Interactions, Drug Load, and Adverse Effects.”

Drug interactions present a special challenge to transitional polytherapy in the conversion of monotherapy to monotherapy. The AEDs are classified as CYP enzyme inducers, enzyme inhibitors, or enzyme neutral drugs. Most clinically relevant CYP interactions occur with isozyme subtype 3A4, although important inhibitory interactions also commonly occur at isozymes 2C9 and 2C19. Similar mechanisms of interaction can occur with induction or inhibition of other hepatic metabolic pathways, especially glucuronic acid conjugation. The following discussion pertains most to CYP level interactions but is also generally relevant to other enzymatic systems.

AEDs that are themselves inducers or inhibitors have an effect on other drugs; specifically, AED inducers or inhibitors may increase or decrease another drug’s metabolism, while enzyme neutral AEDs have no effect on the metabolism of other drugs. AEDs that have enzyme induction and inhibition properties have such effects on other AEDs as well as non-AED drugs used for treatment of other concurrent diseases, such as anticoagulants, antihypertensives, cholesterol-lowering drugs, and oral contraceptives. Also, the metabolism of some AEDs, even those that are themselves enzyme-neutral, may be impacted by other drugs that are inducers or inhibiters. Drug interactions may also occur when adding a new drug or when discontinuing a drug that the patient has been on for an extended period. For example, phenytoin is an enzyme inducer that increases the metabolism of many other drugs. During transitional polytherapy with phenytoin and another inducible drug, when phenytoin is added, the metabolism of the other inducible drug is increased, as is its serum clearance, so the serum concentration of the inducible drug decreases. However, if phenytoin and another inducible drug is being used in combination and phenytoin is removed, the elimination of the other inducible drug will be reduced, and it will instead accumulate and the serum concentration of the inducible drug will increase. This could lead to dangerous complications such as bleeding when the inducible co-medication involved is an anticoagulant such as warfarin. The opposite is true for an AED that is an enzyme inhibitor, such as valproic acid, topiramate, or oxcarbazepine. These enzyme inhibiting AEDs may result in reduced clearance and subsequent accumulation, leading to increased serum concentrations of a co-medication. While most of the focus of drug-drug interactions is on pharmacokinetic interactions of enzyme induction or inhibition, there are some other drugs that have pharmacodynamic interactions. A pharmacodynamic interaction probably occurs at the receptor site. For example, the addition of lamotrigine to the drug regimen of a patient taking carbamazepine may result in a higher incidence of CNS side effects. However, lamotrigine does not interfere with the metabolism of either carbamazepine or its active 10-11 diepoxide metabolite.

AEDs have very different clinical pharmacology and clinical pharmacokinetic profiles. It is important to review these profiles in evaluating rates and methods of transitional polytherapy from monotherapy to monotherapy.

## CLINICAL PHARMACOLOGY OF AEDS

A complete review of clinical pharmacology of the AEDs is beyond the scope of this paper. However, factors such as the route of elimination, half-life, and potential for drug interactions are important factors to consider in transitional polytherapy. These factors will influence the rate of dosage titration and withdrawal.

### Carbamazepine (Tegretol, Tegretol-XR, Carbatrol, Others)

The principle mechanism of action of carbamazepine is thought to relate to blockade of voltage-dependent sodium channels. Carbamazepine is eliminated by hepatic metabolism and is metabolized to an active metabolite, 10-11 carbamazepine epoxide [2]. The concentration of the parent drug and the metabolite may vary independently. Carbamazepine is unique in that it induces its own metabolism and the half-life of carbamazepine becomes shorter with continued dosing. The metabolism of carbamazepine may be induced or inhibited, principally by CYP 3A4, and carbamazepine will induce the metabolism of other AEDs and non-AED drugs. A limitation of carbamazepine is its association with rare but severe idiosyncratic adverse effects such as fatal hepatic injury or Stevens Johnson Syndrome (SJS) [11,17]. Patients of Asian ancestry may be particularly vulnerable to evolution of SJS, and it is recommended by FDA in the United States that patients of Han Chinese ancestry in particular, and others of Asian ancestry who commonly express this genotype, receive preliminary testing for the HLA-B*1502 susceptibility allele for SJS prior to initiating carbamazepine therapy [15].

### Felbamate (Felbatol)

Felbamate is also presumed to have a major mechanism of action of voltage-dependent sodium channel blockade, but it also blocks glutamatergic NMDA receptors [33]. The association of aplastic anemia and hepatotoxicity with felbamate have restricted the use of this AED to those with brittle, refractory epilepsy [34]. Felbamate is eliminated renally and hepatically. The metabolism may be inhibited and induced. Felbamate will inhibit the metabolism of some drugs (eg, phenytoin, valproic acid, carbamazepine epoxide) and induce others (eg, carbamazepine).

### Gabapentin (Neurontin, Others)

Gabapentin binds to the alpha2—delta1 subunit of the presynaptic calcium channel, modulating neurotransmitter release [45]. Gabapentin is actively absorbed. It binds to an L-amino protein carrier system in the gut and this system may become saturated. Therefore, the bioavailability of gabapentin decreases as the dose increases sometimes necessitating more frequent dosing. Gabapentin is eliminated renally, and the clearance of gabapentin correlates with the creatinine clearance [38].

### Lacosamide (Vimpat)

Lacosamide is a novel AED with a presumed mechanism of action related to slow sodium channel inactivation, possibly to modulation of collapsin response mediator protein-2 (CRMP-2) binding that modulates neutrophic factors [10]. This AED is rapidly and completely absorbed, reaches a peak concentration in one hour with a 13-hour elimination half-life, and has a linear concentration curve up to dosing of 800 mg/day [10]. Lacosamide has minor metabolism to an inactive O-desmethyl metabolite and is cleared renally. No significant CYP induction/inhibition, nor any drug interaction with other AEDs or common prescription drugs have been reported.

### Lamotrigine (Lamictal)

The principle mechanism of action for lamotrigine is blockage of voltage gated sodium channels [16]. Lamotrigine is eliminated almost completely by Phase II hepatic metabolism. While the half-life of lamotrigine is around 22 hours in drug naïve patients, indicating that once-a-day dosing would be feasible, the metabolism may be induced or inhibited [16]. The half-life of lamotrigine in patients taking enzyme inducers is around 12 hours [21]. The clearance of lamotrigine is significantly reduced in patients taking valproic acid, due to inhibition of lamotrigine’s glucuronidation by valproate [51]. Unless the dose of lamotrigine is very slowly increased, the use of valproic acid and lamotrigine together is associated with an increase in the incidence of skin rash. While the metabolism of lamotrigine may be induced or inhibited, it does not alter the metabolism of other drugs. Oral contraceptives have been reported to decrease the serum concentration of lamotrigine, potentially rendering it less effective and requiring dose supplementation [39]. A similar phenomena of female hormones lowering lamotrigine’s concentration is also seen during pregnancy, as the serum concentration of lamotrigine may decrease as pregnancy progresses toward full term, then rises again in the postpartum state [47]. The dose of lamotrigine must be increased slowly because of the increased incidence of skin rash with a rapid dosage titration.

### Levetiracetam (Keppra)

Levetiracetam binds the presynaptic SV2A synaptic vesicle protein, which appears to be its principle mechanism of action [31,46]. Most of levetiracetam is eliminated renally with minor elimination *via* hydrolysis that does not involve liver enzymes [1]. The metabolism of levetiracetam is not induced or inhibited, and levetiracetam does not interact with other drugs. The dose of levetiracetam may need to be reduced in patients with renal impairment. Due to its generally excellent tolerability, levetiracetam dosage can generally be increased rapidly.

### Oxcarbazepine (Trileptal)

Oxcarbazepine is a prodrug and is rapidly converted to a monohydroxylated derivative (MHD) which is active [42]. MHD is eliminated by glucuronide conjugation or hydroxylation as well as renally. Patients with renal dysfunction have a decreased clearance of MHD. Although this drug is structurally and mechanistically related to carbamazepine, there is no auto induction [40]. While the metabolism of oxcarbazepine to MHD is not induced or inhibited, the further metabolism of MHD may be altered by other drugs; in particular, the metabolism of MHD is inducible at CYP 3A4 [42]. Also, oxcarbazepine or MHD may interact with other drugs; an important interaction is the possibility of reduced hormonal contraceptives concentrations in women of child bearing potential receiving oxcarbazepine [14]. Oxcarbazepine may have favorable impact upon mood in patients with epilepsy [32].

### Phenobarbital (Luminal)

Phenobarbital is the oldest AED still in active use and remains the most commonly used AED worldwide, particularly in the treatment of neonatal seizures where it remains a drug of choice given extensive experience with its use in this patient population [6,28]. However, the use of Phenobarbital has waned substantially in the United States and Europe given its inferior tolerability to other older drugs and the newer AEDs [28]. The main mechanism of action of phenobarbital is related to increased duration of opening of the chloride ionophore by binding the alpha subunit of the GABA-A receptor complex [28]. Phenobarbital has both hepatic and renal elimination. The half-life of phenobarbital ranges from 72 to 125 hours, necessitating very slow dosage adjustment. The metabolism of phenobarbital may be inhibited or induced. Phenobarbital is a potent CYP enzyme inducer.

### Phenytoin (Dilantin, Phenytek, Others)

The main mechanism of action for phenytoin is blockade of voltage gated sodium channels. Phenytoin has very complex pharmacokinetics displaying saturable absorption, saturable metabolism (Michaelis-Menton kinetics), and high protein binding [23]. This drug is eliminated primarily by hepatic metabolism. However, the half-life is concentration dependant due to saturable metabolism. Saturable metabolism means that small changes in dose may result in significantly larger increases in serum concentration. The metabolism of phenytoin may be inhibited or induced, and phenytoin induces the metabolism of other drugs.

### Pregabalin (Lyrica)

Like its structural analog gabapentin, the main mechanism of pregabalin appears to be related to binding at the alpha2—delta1 subunit of the presynaptic calcium channel, modulating neurotransmitter release [44].

Although this drug is structurally related to gabapentin, the pharmacokinetics of pregabalin are linear because pregabalin does not display the saturable absorption shown by gabapentin [12]. Pregabalin is completely eliminated renally and is not protein bound. Patients with renal impairment will have a decreased pregabalin clearance. There are no drug interactions reported with pregabalin. The dose of pregabalin may be rapidly increased as tolerated, although many patients require slower titration due to CNS adverse effects.

### Rufinamide (Banzel)

This triazole derivative is structurally unrelated to currently marketed AEDs, and its major mechanism of action appears to be related to prolonged inactivation of sodium channels [3]. The extent of rufinamide absorption decreases with higher dosing. The drug is extensively metabolized by carboxylase enzymatic degradation to an inactive carboxylic acid, and renally cleared. Rufinamide is a weak CYP3A4 inducer, so it may impact metabolism of other AEDs and medications cleared through CYP3A4 including hormonal contraceptives. Plasma half-life is 6-10 hours. Rufinamide may increase phenytoin concentrations to some degree, but has no significant effects on other AEDs. Valproate decreases rufinamide clearance in children by up to 70%, so titration of either drug should be adjusted accordingly when rufinamide and valproate are co-administered.

### Tiagabine (Gabatril)

The presumed mechanism of action is related to its activity as a potent and selective blocker of the GAT-1 GABA transporter, thereby inhibiting reuptake of GABA into presynaptic neurons and prolonging its availability synaptically [22,49]. Tiagabine is eliminated by hepatic metabolism, which can be induced or inhibited. However, this drug is not reported to affect the metabolism of other drugs. Tiagabine is highly protein bound. The dose of tiagabine must be slowly increased because of increased side effects with rapid dosage titration.

### Topiramate (Topamax)

The main mechanism of action of topiramate is thought to be related to voltage gated sodium ion channel blockade, but it also has a variety of other synaptic and non-synaptic effects, including blockade of glutamatergic non-NMDA kainate/AMPA receptors, facilitation of GABAergic neurotransmission, and mild inhibition of carbonic anhydrase [41]. Topiramate has both renal and liver elimination. However, the renal elimination predominates. In patients with hepatic dysfunction, renal elimination may increase [19]. Patients with decreased renal function will have a decreased clearance of topiramate. Enzyme inducers increase clearance of topiramate. Topiramate inhibits the metabolism of some patients taking phenytoin but not all. The interaction appears to occur in those patients who are at the point of saturating their phenytoin metabolism [23]. The dose of topiramate must be slowly increased because the incidence of side effects may increase with a rapid dosage titration, especially when topiramate is used in adjunctive therapy with other AEDs. The propensity for topiramate adverse effects with monotherapy use appears to be substantially lower than when topiramate is used in adjunctive therapy settings [36].

### Valproic acid (Sodium Divalproex) (Depakene, Depakote, Depakote-ER)

Valproate also has a predominant mechanism of action related to blockade of voltage gated sodium channels, but there is also evidence for additional mechanisms including facilitation of GABAergic neurotransmission and inhibition of glutamatergic neurotransmission *via* NMDA receptor inhibition [30]. The rate of absorption of valproic acid depends on the formulation; the sodium salt with enteric coating (Depakote) is well absorbed, while the generic valproic acid formulation (Depakene) is poorly absorbed and often causes nausea, especially during initial titration. Valproic acid is metabolized by the liver by Phase II metabolism to multiple metabolites, one of which may be liver toxic [37]. Valproic acid is highly protein bound. However, the binding saturates at concentrations around 90 mcg/mL. This increases the free fraction, which increases the clearance. Thus, the percent increase in total concentration will be less than the percent increase in dose.

### Zonisamide (Zonegran)

The principle mechanism for zonisamide again appears to be related to blockade of voltage-dependent sodium channels, limiting sustained neuronal burst firing, but it also has several additional mechanisms, including inhibition of low-threshold T-type calcium channels and weak inhibition of carbonic anhydrase [5]. About 70% of zonisamide is metabolized in the liver and about 30% is eliminated renally [24]. While the metabolism of zonisamide may be induced or inhibited, zonisamide does not alter the clearance of other drugs. The dose of zonisamide should be slowly increased to reduce the incidence of side effects, especially CNS side effects.

While the need for transitional polytherapy in the conversion of monotherapy to monotherapy has been established clinically, there are few published papers on how this should be done. While some have considered this to be more art than science, a recent panel of experts was convened to establish standard recommendations to guide all clinicians in transitional polytherapy.

## SPECTRA CONSENSUS PANEL RECOMMENDATIONS

There is very little published data to assist the clinician in determining the rate of upward titration and downward taper of AEDs during the process of transitional polytherapy from monotherapy to monotherapy. A panel of neurologists and clinical pharmacists who specialize in the care of patients with epilepsy was assembled to participate in a therapy conversion Delphi study called SPECTRA (Study by a Panel of Experts: Considerations for Therapy Replacement in Antiepileptics). The goal of SPECTA was to develop a practical guide on AED monotherapy conversion, and the consensus recommendations were recently published [45].

The process involved two web-based surveys and one live consensus panel. In the first web-based survey, panel members were asked questions about therapy conversion in adults. The second web-based survey responded to the answers from the first survey. The questionnaires used the Delphi technique to elicit individual responses and facilitate the experts in refining their views as the group proceeded to agreement. Delphi is a group facilitation technique that seeks to obtain consensus on the opinions of experts through a series of structured questionnaires commonly referred to as rounds. This technique maintains anonymity, controls feedback, and provides statistically based responses. After the second web-based survey, the group was convened to reach consensus on the rate of tapering of the old AED, the rate of titration of the new AED, and the usefulness of drug level monitoring. During the third round, panel members reviewed each statement and voted anonymously using an automated response system (ARS). The response options ranged from 1 to 5, where 1 = strongly disagree, 2 = disagree, 3 = neutral, 4 = agree, and 5 = strongly agree. Generally, a mean score of 4.0 (agree) was required to indicate that consensus had been reached. The panel did not feel that sufficient experience or literature existed to provide recommendations regarding the approved AED pregabalin (Lyrica), nor did they consider lacosamide (Vimpat) or rufinamide (Banzel) since these drugs were investigational at the time the panel was convened.

The panel reached a consensus for the following recommendations for a general taper and titration.

### Starting a Taper

The panel recommended that the old baseline AED be tapered after a presumably efficacious dose was reached with the new planned AED. However, if a patient experienced significant adverse effects during the conversion, the taper of the old baseline AED may be started sooner.

### Tapering

**Fig. (2):**
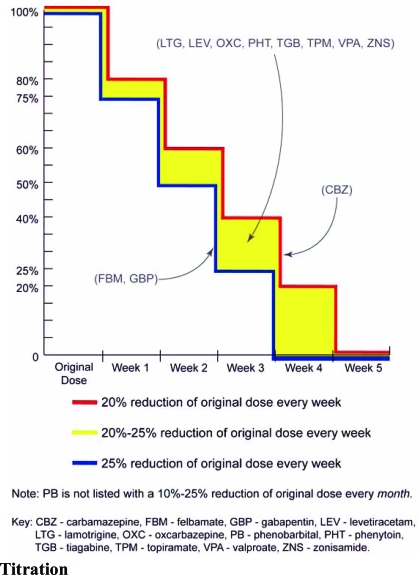
**General Tapering Method.** Note that phenobarbital, with a 10-25% reduction of original dose every month, is not illustrated. CBZ, carbamazepine; FBM, felbamate; GBP, gabapentin; LEV, levetiracetam; LTG, lamotrigine; OXC, oxcarbazepine; PB, phenobarbital; PHT, phenytoin; TGB, tiagabine; TPM, topiramate; VPA, valproate; ZNS, zonisamide. (reproduced with kind permission from Elsevier, Inc. St. Louis *et al*; Conversions between monotherapies in epilepsy: expert consensus. *Epilepsy and Behavior* 2007; 11: 224).

The panel recommended slower tapering and smaller reductions for persons who are seizure free and licensed to drive for all agents. However, in 10 out of 12 drugs reviewed, a faster taper was recommended if a patient experienced significant adverse events. The exceptions were carbamazepine and valproic acid. The panel only recommended a slower taper if patients experienced inadequate seizure control when receiving tiagabine and topiramate. General drug specific tapering strategies are depicted in (Fig. **2**), and drug specific alterations to the general tapering strategy are summarized in Table **1**.

### Titration

After reviewing the package inserts titration schedules, the panel agreed with this method for lamotrigine, tiagabine, and zonisamide. For the nine other AEDs, they recommended additional methods to assist with titration and tapering processes. Factors such as enzyme inducers and advanced age modified titration schemes.

The panel reached a consensus on the taper and titration of individual AEDs. Drug specific general titration strategies are summarized in Table **2**, and alterations to the general titration strategies are shown in Table **3**.

### Carbamazepine

The general taper is 20% of the original dose every week. A faster taper was recommended for patients with reduced liver function and patients being converted to oxcarbazepine. A larger reduction in dose was recommended for patients with significant adverse effects. A smaller reduction in dose was recommended for patients with inadequate seizure control.

The initial dose recommended for carbamazepine was 200 mg/day with a dosage increase of 200 mg every 7 days. After a total daily dose of 400 to 800 mg/day was achieved, the panel would use drug levels to determine the next course of action. A faster titration was recommended in patients with inadequate seizure control or for patients who were converting from oxcarbazepine. A slower titration was recommended for elderly patients, patients with reduced liver function, patients with mild adverse effects, or patients converting from felbamate. A larger increase in dose was recommended for inadequate seizure control, and a smaller increase in dose was recommended for elderly patients.

### Felbamate

The panel recommended that the general taper for felbamate be 25% of the original dose every week. The taper could be faster in patients with reduced liver function and in patients with significant adverse effects. The percent dose reduction could be larger in patients with reduced liver function and smaller in patients with inadequate seizure control.

The recommended starting dose was 600 mg/day. The dose could be increased by 300 mg every 7 days to a target dose of 1200 to 1800 mg/day. A slower titration was recommended for patients with mild side effects. The panel felt that this AED should only be used by epileptologists. They did not want to encourage the use of felbamate in the elderly or in patients with reduced liver function and, therefore, did not offer any suggestions for these groups.

### Gabapentin

The general taper recommended for gabapentin was 25% of the original dose every week. A faster taper would be acceptable for patients with renal insufficiency or patients with significant adverse effects. A larger dose reduction would be acceptable for patients with significant adverse effects, and a smaller reduction would be acceptable for patients with inadequate seizure control.

As the initial dose of gabapentin, 600 to 900 mg/day was recommended, with an increase of 600 to 900 mg every 7 days to a total dose of 1800 to 2700 mg. A faster titration was recommended for inadequate seizure control. A slower titration was recommended for elderly patients, patients with renal insufficiency, and patients with mild adverse effects. A larger dose increase was recommended for patients with inadequate seizure control.

### Lamotrigine

A reduction of 20% to 25% of the original dose was recommended as the general taper of lamotrigine. A faster taper was recommended for patients with reduced liver function, patients taking valproic acid, and patients with significant adverse effects. A larger dosage reduction was endorsed for patients with reduced liver function and for patients converting to valproic acid monotherapy, while a smaller reduction in dose was endorsed for patients with inadequate seizure control.

The panel accepted the package insert recommendations for the starting dose and for the dosing interval. After a dose of 200 to 500 mg/day was reached, the panel recommended that levels be used to determine the next course of action. The panel did not recommend any faster rate of titration but did recommend a slower rate of titration for patients with mild adverse effects and for patients who were converting from valproic acid. A larger dosage increase was recommended for patients with inadequate seizure control, and a smaller dosage increase was recommended for patients with reduced liver function. The panel referred clinicians to the package insert for dosage adjustments for patients converting from valproic acid or enzyme-inducing AEDs.

### Levetiracetam

The panel recommended that a general taper of levetiracetam begin with a 20% to 25% reduction in the original dose every week. The taper may be faster in patients with renal insufficiency or significant adverse effects. The percent reduction may be larger in patients with significant adverse effects or renal insufficiency and smaller in patients with inadequate seizure control.

A starting dose of 500 to 1000 m/day of levetiracetam was recommended. The recommended dosing interval was 500 to 1000 mg every 7 days to a target dose of 1000 to 2000 mg/day. A faster titration was endorsed for patients with inadequate seizure control. A slower titration was recommended for elderly patients, patients with renal insufficiency, and patients with mild adverse effects. A larger dose increase was recommended for patients with inadequate seizure control, and a smaller increase was recommended for elderly patients.

### Oxcarbazepine

The general taper recommended for oxcarbazepine was 20% to 25% of the original dose every week. A faster taper was approved for patients converting from carbamazepine and for patients with significant adverse effects. No recommendations were made for a slower taper. A larger dose reduction was deemed appropriate for patients with significant adverse side effects and for patients converting from carbamazepine monotherapy. A smaller reduction was acceptable for patients with inadequate seizure control.

The recommended starting dose of oxcarbazepine was 300 to 600 mg/day. A 300 mg increase in dose every 7 days was recommended up to a target dose of 900 to 1500 mg/ day. A faster rate of titration was acceptable for patients with inadequate seizure control and for patients converting from carbamazepine, while a slower titration was acceptable for patients with reduced liver function. A larger dosage increase was agreed upon for patients with inadequate seizure control and for those patients who are converting from carbamazepine, while a smaller increase was recommended for patients with reduced liver function.

### Phenobarbital

The general taper that was recommended for phenobarbital was 10% to 25% of the original dose every month. A faster taper was recommended for patients on phenobarbital less than 1 month and for those with significant adverse effects. No recommendations were made for a slower taper. A larger reduction in dose was recommended for patients with significant adverse effects, while a smaller reduction was recommended for patients with inadequate seizure control.

The panel did not make any recommendations for phenobarbital titration, as switching a patient to Phenobarbital was not encouraged.

### Phenytoin

A 20% to 25% reduction of the original dose every month was recommended as a general taper for phenytoin. A faster taper was recommended for patients with reduced liver function or significant adverse effects. A larger reduction in dose was recommended for patients with reduced liver function. No recommendations were made for a slower taper or for a smaller reduction in dose.

The recommended starting dose of phenytoin was 3 to 5 mg/kg/day. The recommended dosage adjustments were 30 mg/day for steady state levels >12 mcg/mL, 50 mg/day for steady state levels between 7 and 12 mcg/mL, and 100 mg/day for steady state levels <7 mcg/mL [35]. The target dose was dependant on the drug level.

### Tiagabine

The general taper recommended for tiagabine was 20% to 25% of the original dose every week. A faster taper may be appropriate for patients with reduced liver function or patients with significant adverse effects, and a slower taper may be appropriate for patients with inadequate seizure control. The panel recommended a larger reduction in dose for patients with reduced liver function but did not make any recommendations for smaller reductions in dose.

The panel accepted the package insert suggestions for dosage titration. A faster titration was recommended for patients with inadequate seizure control, and a slower titration was recommended for elderly patients, patients with reduced liver function, and patients with mild adverse effects. The panel recommended a larger dose increase in patients with inadequate seizure control, and a smaller dosage increase in elderly patients and patients with reduced liver function.

### Topiramate

A 20% to 25% reduction of the original dose every week was recommended as the general taper for topiramate. A faster taper was recommended for patients with renal insufficiency or significant adverse effects. A slower taper was recommended for patients with inadequate seizure control. A larger reduction in dose was recommended for patients with renal insufficiency, and a smaller reduction in dose was recommended for patients with inadequate seizure control.

The initial dose that was recommended was 25 to 50 mg/day with dosage increases of 25 to 50 mg every 7 days up to 100 to 200 mg/day. Faster titration was recommended for patients with inadequate seizure control, and a slower titration was recommended for patients with renal insufficiency. A larger increase in dose was recommended for patients with inadequate seizure control, and a smaller increase in dose was recommended for elderly patients and patients with renal insufficiency.

### Valproate (Valproic Acid, Sodium Divalproex)

The general taper that was recommended for valproate was 20% to 25 % of the original dose every week. A faster taper and a larger dosage reduction were recommended for patients with reduced liver function. No recommendations were made for slower tapers or smaller dosage reduction.

For the starting dose of valproate, 500 to 1000 mg/day was recommended. Dosage increases of 250 to 750 mg every 7 days were recommended up to a dose of 1000 to 2000 mg/day. Blood levels would then determine the next course of action. The panel made no recommendation for faster dosage titration but recommended a slower titration in elderly patients and patients with mild adverse effects. The panel recommended a larger increase in dose for patients with inadequate seizure control and a smaller increase in dose for elderly patients.

The panel also agreed that the adverse effects of valproate are final-dose related and are not usually titration related. The panel concluded that the target concentration of valproate for general seizures is on the lower end of the target concentrations.

### Zonisamide

A 20% to 25% reduction of the original dose every week was recommended as the general taper for zonisamide. A faster titration and larger increase and dosage for patients with inadequate seizure control. No recommendations were made for a slower taper or for a smaller dosage reduction.

The panel accepted the package insert method for initiating and titrating zonisamide. The panel recommended a faster titration and larger increase in dosage reduction for patients with inadequate seizure control and a slower titration and a smaller dosage increase for elderly patients.

### Blood Level Monitoring

The panel also discussed drug level monitoring during transitional polytherapy from monotherapy to monotherapy, recommending drawing a drug level for the new AED before withdrawing the old AED. The panel also recommended obtaining a level of the new AED once the patient is on monotherapy to be used as a baseline or benchmark level to which future comparisons could be made when necessary. Examples of scenarios in which a previously efficacious, steady-state AED dosing and concentration could be threatened include problems with patient adherence, new drug-drug interactions, new medical illness, or pregnancy.

## CONCLUSION

The concept of monotherapy for the treatment of epilepsy is well established. However, at least 50% of patients fail therapy with the first AED. Conversion from one AED to another is complex. Generally, sudden withdrawal of initial AED therapy is discouraged unless a patient has experienced serious adverse effects. In most instances when patients are changing from one AED to another, there will be a period of transitional polytherapy during the conversion from monotherapy to monotherapy. This process is complicated by drug interactions and complex AED pharmacokinetics.

Until now, physicians have had to rely on their own clinical experience to determine the rate of AED tapering and titration. The SPECTRA report utilized the well-validated Delphi technique to achieve consensus from practicing epileptologists and clinical pharmacists on tapering and titrating AEDs. Now there is a set of guidelines that clinicians can use to compare their clinical experiences and aid them in the period of transitional polytherapy with antiepileptic drugs from monotherapy to monotherapy.

## Figures and Tables

**Table 1:** **Summary of Drug Specific Alterations to General Tapering Methods.** Antiepileptic Drugs (AEDs) are listed alphabetically. The SPECTRA Panel reached consensus for modification of the usual general tapering method for each drug in certain situations, and recommended faster or slower tapering, by larger or smaller dose decrements, in the situations listed in each column for each individual AED. CBZ, carbamazepine; FBM, felbamate; GBP, gabapentin; LEV, levetiracetam; LTG, lamotrigine; OXC, oxcarbazepine; PB, phenobarbital; PHT, phenytoin; TGB, tiagabine; TPM, topiramate; VPA, valproate; ZNS, zonisamide. (modified and reproduced with kind permission from Elsevier, Inc. St. Louis et al; Conversions between monotherapies in epilepsy: expert consensus. *Epilepsy and Behavior* 2007; 11: 226).

AED	Faster Taper	Slower Taper	Larger Dose Decrements	Smaller Dose Decrements
1. CBZ	Impaired liver function Conversion to OXC		Significant adverse effects	Inadequate seizure control
2. FBM	Impaired liver function Significant adverse effects		Inadequate seizure control	Impaired liver function
3. GBP	Renal insufficiency Significant adverse effects		Significant adverse effects	Inadequate seizure control
4. LTG	Impaired liver function Converting to VPA Significant adverse effects		Impaired liver function Converting to VPA	Inadequate seizure control
5. LEV	Renal insufficiency Significant adverse effects		Significant adverse effects Renal insufficiency	Inadequate seizure control
6. OXC	Converting to CBZ Significant adverse events		Significant adverse effects Converting to CBZ	Inadequate seizure control
7. PB	On PB <1 month Significant adverse effects		Significant adverse effects	Inadequate seizure control
8. PHT	Impaired liver function Significant adverse effects		Impaired liver function	
9. TGB	Impaired liver function Significant adverse effects	Inadequate seizure control	Impaired liver function	
10. TPM	Renal insufficiency Significant adverse effects	Inadequate seizure control	Renal insufficiency	Inadequate seizure control
11.VPA	Impaired liver function		Impaired liver function	
12. ZNS	Significant adverse effects		Inadequate seizure control	

**Table 2:** **Summary of Drug Specific General Titration Methods.** The SPECTRA panel provided consensus recommendations for general strategies of titrating each of the antiepileptic drugs (AEDs) shown below. Recommended starting doses, titration schemes, and target doses were agreed upon for each AED. The final column indicates whether or not the panel reached consensus regarding a recommendation for blood level monitoring during titration. CBZ, carbamazepine; FBM, felbamate; GBP, gabapentin; LEV, levetiracetam; LTG, lamotrigine; OXC, oxcarbazepine; PB, phenobarbital; PHT, phenytoin; TGB, tiagabine; TPM, topiramate; VPA, valproate; ZNS, zonisamide. (reproduced and modified with kind permission from Elsevier, Inc. St. Louis et al; Conversions between monotherapies in epilepsy: expert consensus. *Epilepsy and Behavior* 2007; 11: 225).

AED	Starting Dose	Interval (Increase)	Target Daily Dose	Monitor Blood Level During Titration
1. CBZ	200 mg/day	200 mg every 7 days	400-800 mg/day^a^	Yes
2. PBM	600 mg/day	300 mg every 7 days	1200-1800 mg/day	No consensus
3. GBP	600-900 mg/day	600-900 mg every 7 days	1800-2700 mg/day	No consensus
4. LTG^b^ Package insert Existing AED is CBZ, PHT, PB or primidone Existing AED is VPA Existing AED is not CBZ, PHT, PB VPA or primidone	50 mg/day 25 mg every other day 25 mg every day	50 mg every day for 2 weeks. 100 mg every day for weeks 3 and 4; then 100 mg every week 25 mg every other day for 2 weeks. 25 mg every day for weeks 3 and 4. 50 mg every day for week 5. 25-50 mg every 1-2 weeks. 50 mg every day for weeks 3 and 4. 100 mg every day every 7 days	200-500 mg/day 100-200 mg/day 300-500 mg/day	No consensus No consensus No consensus
5. LEV	500-1000 mg/day	500-1000 mg every 7 days	1000-2000 mg/day	No consensus
6. OXC	300-600 mg/day	300 mg every 7 days	900-1500 mg/day	No consensus
7. PB^c^				
8. PHT	3-5 mg/kg/day	30 mg/day if steady state Level >12 µg/mL: 50 mg /day if steady state Level 7-12 µg/mL 100 mg/day if steady state Level <7 µg/mL	Therapeutic blood level	Yes
9. TGB Package insert Existing drug is enzyme-inducer Existing drug not an enzyme-inducer^d^	4 mg/day	4 mg every 7 days for 4 weeks. 4-8 mg every 7 days in weeks 5 and 6.	32-56 mg/day in 2-4 divided doses	No consensus
10. TPM	25-50 mg/day	25-50 mg every 7 days	100-200 mg/day	No consensus
11. VPA	500-1000 mg/day	250-750 mg every 7 days	1000-2000 mg/day^e^	Yes
12. ZNS Package insert	100 mg/day	100 mg every 2 weeks	300-400 mg/day	No consensus

a For CBZ, use higher end of target daily dose if converting from an enzyme inducer.

b For LTG, titration schemes outlined are taken from US packaging insert guidelines: international clinicians are advised to consult package insert recommendations and approvals in countries, which may differ from US standards.

c Titration with PB is not encouraged (the SPECTRA panel decided not to provide specific recommendations regarding phenobarbital, reflecting a general discouragement for use of phenobarbital in modern epilepsy treatment).

d Following a given dose of TGB, the estimated plasma concentration in non-induced patients is more than twice that in patients receiving enzyme-inducing  agents. Use in non-induced patients requires lower doses of TGB. These patients may also require a lower titration of TGB. These patients may also require a slower titration of TGB compared with induced patients.

e For VPA, use lower end of range for generalized seizures.

**Table 3:** **Summary of Drug Specific Alterations to General Titration Methods.** Antiepileptic Drugs (AEDs) are listed alphabetically. The SPECTRA Panel reached consensus for modification of the usual general titration schemes for each drug in certain situations, and recommended faster or slower tapering, by larger or smaller dose decrements, in the situations listed in each column for each individual AED. CBZ, carbamazepine; FBM, felbamate; GBP, gabapentin; LEV, levetiracetam; LTG, lamotrigine; OXC, oxcarbazepine; PB, phenobarbital; PHT, phenytoin; TGB, tiagabine; TPM, topiramate; VPA, valproate; ZNS, zonisamide. (reproduced and modified with kind permission from Elsevier, Inc. St. Louis et al; Conversions between monotherapies in epilepsy: expert consensus. *Epilepsy and Behavior* 2007; 11: 226).

AED	Faster Titration	Slower Titration	Larger Dose Increments	Smaller Dose Increments
1. CBZ	Inadequate seizure control Conversion from OXC	Elderly patient Impaired liver function Mild adverse effects Converting from FBM	Inadequate seizure control	Elderly patient
2. FBM^a^		Mild adverse effects	Inadequate seizure control	Impaired liver function
3. GBP	Inadequate seizure control	Elderly patient Renal insufficiency Mild adverse effects	Inadequate seizure control	
4. LTG^b^		Mild adverse effects Converting to VPA^c^	Inadequate seizure control	Impaired liver function
5. LEV	Inadequate seizure control	Elderly patient Renal insufficiency Mild adverse effects	Inadequate seizure control	Elderly patient
6. OXC	Converting from CBZ Inadequate seizure control	Impaired liver function	Inadequate seizure control Converting from CBZ	Impaired liver function
7. PB^d^				
8. PHT^e^				
9. TGB	Inadequate seizure control	Elderly patient Impaired liver function Mild adverse effects	Inadequate seizure control	Impaired liver function Elderly patient
10. TPM	Inadequate seizure control	Renal insufficiency	Inadequate seizure control	Elderly patient Renal insufficiency
11. VPA		Elderly patient Mild adverse effects^f^	Inadequate seizure control	Elderly patient
12. ZNS	Inadequate seizure control	Elderly patient	Inadequate seizure control	Elderly patient

*Note:* All references to package insert refer to US packaging: international clinicians are encouraged to consult packaging information and recommended prescribing practices in their own countries, which may vary substantially from US standards.

a FBM should not be used by a nonepileptologist: should not be used in elderly patients: and should not be used in patients with impaired liver function.

b Should refer to package insert when converting from PHT.

c Should refer to package insert when converting from VPA.

d Titration with PB is not encouraged (the SPECTRA panel decided not to provide specific recommendations regarding phenobarbital, reflecting a general discouragement for use of phenobarbital in modern epilepsy treatment).

e No alterations to the general titration method are recommended.

f Adverse events are final dose related, not titration related.
